# Supplementary material for: Survival Benefit of Surgical Treatment for Elderly Patients with Intrahepatic Cholangiocarcinoma: A Retrospective Cohort Study in the SEER Database by Propensity Score Matching Analysis
Source: Curr Oncol. 2023 Feb 22;30(3):2642–52. doi: 10.3390/curroncol30030201 (PMC10047145; doi:10.3390/curroncol30030201)
Supplement: Supplementary file 1 [file curroncol-30-00201-s001.zip › curroncol-2215731-supplementary.pdf]

TableS1 The proportion of TNM stages for TNM I-IV,unmatched TNM I-IIIB and matched TNM I-IIIB for ICC patients.

TableS2 Characteristics of ICC patients above 65 years old before and after propensity score matching

TableS3 Characteristics of ICC patients above 70 years old before and after propensity score matching

TableS4 Characteristics of ICC patients above 75 years old before and after propensity score matching

TableS5 Estimated 3-month and 6-month survival rates of surgery and non-surgery groups for M0 stage ICC patients in different age stratification

TableS6 Estimated 3-month and 6-month survival rates of surgery and non-surgery groups for N0 stage ICC patients in different age stratification

TableS7 Estimated 3-month and 6-month survival rates of surgery and non-surgery groups for N1 stage ICC patients in different age stratification

TableS8 Univariate and multivariate cox results table for above 60 years old patients of ICC for overall survival

TableS9 Univariate and multivariate cox results table for above 65 years old patients of ICC for overall survival

TableS10 Univariate and multivariate cox results table for above 70 years old patients of ICC for overall survival

TableS11 Univariate and multivariate cox results table for above 75 years old patients of ICC for overall survival

TableS12 Univariate and multivariate cox results table for above 60 years old patients of ICC for cancer-specific survival

TableS13 Univariate and multivariate cox results table for above 65 years old patients of ICC for cancer-specific survival

TableS14 Univariate and multivariate cox results table for above 70 years old patients of ICC for cancer-specific survival

TableS15 Univariate and multivariate cox results table for above 75 years old patients of ICC for cancer-specific survival

TableS16 Estimated 2-year survival rates of surgery and non-surgery groups for ICC patients above 80 years old

TableS17 Estimated 3-month and 6-month survival rates of surgery and non-surgery groups for ICC patients above 80 years old

FigureS1 Flowchart of Patient Selection.

FigureS2 Forest plot and summary statistics of the surgery influence on intrahepatic cholangiocarcinoma incidence for OS in ICC.

FigureS3 Forest plot and summary statistics of the surgery influence on intrahepatic cholangiocarcinoma incidence for CSS in ICC.

TableS1 The proportion of TNM stages for TNM I-IV,unmatched TNM I-IIIB and matched TNM I-IIIB for ICC patients.

TNM Distribution	Non-Surgery	Surgery	P-value	
TNM I-IV			<0.001	1.026
IA	54 (5.6)	79 (21.1)		
IB	85 (8.8)	48 (12.8)		
II	212 (21.9)	107 (28.5)		
IIIA	8 (0.8)	6 (1.6)		
IIIB	173 (17.8)	109 (29.1)		
IV				
Unmatched TNM I-IIIB			<0.001	0.357
IA	54 (10.2)	79 (22.6)		
IB	85 (16.0)	48 (13.8)		
II	212 (39.8)	107 (30.7)		
IIIA	8 (1.5)	6 (1.7)		
IIIB	173 (32.5)	109 (31.2)		
Matched TNM I-IIIB			0.398	0.181
IA	31 (12.4)	46 (18.5)		
IB	37 (14.9)	33 (13.3)		
II	97 (39.0)	85 (34.1)		
IIIA	6 (2.4)	5 (2.0)		
IIIB	78 (31.3)	80 (32.1)		

Abbreviations: ICC, intrahepatic cholangiocarcinoma; TNM, tumor node metastasis.

TableS2 Characteristics of ICC patients above 65 years old before and after propensity score matching (continued)

Characteristic	Unmatched<br>ALL<br>(*n*=619)	 Non-surgery<br>(*n*=416)	 Surgery<br>(*n*=253)	 *P*-value	Matched<br>ALL<br>(*n*=388)	 Non-surgery<br>(*n*=194)	 Surgery<br>(*n*=194)	 *P*-value
Age, years	73.7±6.44	74.5±6.84	72.3±5.49	<0.001	72.5±5.46	72.5±5.61	72.6±5.31	0.809
Gender				0.202				1.000
Female	353(52.8%)	228(54.8%)	125(49.4%)		198(51.0%)	99(51.0%)	99(51.0%)	
Male	316(47.2%)	188(45.2%)	128(50.6%)		190(49.0%)	95(49.0%)	95(49.0%)	
Ethnicity				0.912				1.000
White	534(79.8%)	331(79.6%)	203(80.2%)		313(80.7%)	156(80.4%)	157(80.9%)	
Non-white	135(20.2%)	85(20.4%)	50(19.8%)		75(19.3%)	38(19.6%)	37(19.1%)	
Grade				0.014				0.751
Well-moderately	392(58.6%)	228(54.8%)	164(64.8%)		248(63.9%)	122(62.9%)	126(64.9%)	
Poorly-undifferentially	277(41.4%)	188(45.2%)	89(35.2%)		140(36.1%)	72(37.1%)	68(35.1%)	
TNM stage				0.001				0.059
IA	97(14.5%)	42(10.1%)	55(21.7%)		59(15.2%)	21(10.8%)	38(19.6%)	
IB	109(16.3%)	76(18.3%)	33(13.0%)		62(16.0%)	35(18.0%)	27(13.9%)	
II	240(35.9%)	161(38.7%)	79(31.2%)		136(35.1%)	77(39.7%)	59(30.4%)	
IIIA	11(1.64%)	6(1.44%)	5(1.98%)		9(2.32%)	4(2.06%)	5(2.58%)	
IIIB	212(31.7%)	131(31.5%)	81(32.0%)		122(31.4%)	57(29.4%)	65(33.5%)	
Chemotherapy				0.019				0.835
No	386(57.7%)	225(54.1%)	161(63.6%)		239(61.6%)	121(62.4%)	118(60.8%)	
Yes	283(42.3%)	191(45.9%)	92(36.4%)		149(38.4%)	73(37.6%)	76(39.2%)	
Radiotherapy				0.009				1.000
No	550(82.2%)	329(79.1%)	221(87.4%)		331(85.3%)	165(85.1%)	166(85.6%)	
Yes	119(17.8%)	87(20.9%)	32(12.6%)		57(14.7%)	29(14.9%)	28(14.4%)	

Abbreviations: ICC, intrahepatic cholangiocarcinoma; TNM, tumor node metastasis.

TableS3 Characteristics of ICC patients above 70 years old before and after propensity score matching (continued)

Characteristic	Unmatched ALL (*n*=454)	Unmatched Non-surgery (*n*=296)	Unmatched Surgery (*n*=158)	Unmatched *P*-value	Matched ALL (*n*=284)	Matched Non-surgery (*n*=142)	Matched Surgery (*n*=142)	Matched *P*-value
Age, years	76.8±5.45	77.5±5.80	75.5±4.46	<0.001	75.9±4.75	75.8±5.04	76.0±4.46	0.813
Gender				0.077				1.000
Female	237(52.2%)	164(55.4%)	73(46.2%)		137(48.2%)	68(47.9%)	69(48.6%)	
Male	217(47.8%)	132(44.6%)	85(53.8%)		147(51.8%)	74(52.1%)	73(51.4%)	
Ethnicity				0.693				1.000
White	368(81.1%)	242(81.8%)	126(79.7%)		230(81.0%)	115(81.0%)	115(81.0%)	
Non-white	86(18.9%)	54(18.2%)	32(20.3%)		54(19.0%)	27(19.0%)	27(19.0%)	
Grade				0.02				0.713
Well-moderately	261(57.5%)	158(53.4%)	103(65.2%)		178(62.7%)	91(64.1%)	87(61.3%)	
Poorly-undifferentially	193(42.5%)	138(46.6%)	55(34.8%)		106(37.3%)	51(35.9%)	55(38.7%)	
TNM stage				0.015				0.088
IA	68(15.0%)	32(10.8%)	36(22.8%)		43(15.1%)	15(10.6%)	28(19.7%)	
IB	82(18.1%)	58(19.6%)	24(15.2%)		50(17.6%)	29(20.4%)	21(14.8%)	
II	162(35.7%)	111(37.5%)	51(32.3%)		108(38.0%)	60(42.3%)	48(33.8%)	
IIIA	6(1.32%)	5(1.69%)	1(0.63%)		3(1.06%)	2(1.41%)	1(0.70%)	
IIIB	136(30.0%)	90(30.4%)	46(29.1%)		80(28.2%)	36(25.4%)	44(31.0%)	
Chemotherapy				0.004				0.613
No	285(62.8%)	171(57.8%)	114(72.2%)		191(67.3%)	93(65.5%)	98(69.0%)	
Yes	169(37.2%)	125(42.2%)	44(27.8%)		93(32.7%)	49(34.5%)	44(31.0%)	
Radiotherapy				<0.001				1.000
No	373(82.2%)	229(77.4%)	144(91.1%)		255(89.8%)	127(89.4%)	128(90.1%)	
Yes	81(17.8%)	67(22.6%)	14(8.86%)		29(10.2%)	15(10.6%)	14(9.86%)	

Abbreviations: ICC, intrahepatic cholangiocarcinoma; TNM, tumor node metastasis.

TableS4 Characteristics of ICC patients above 75 years old before and after propensity score matching (continued)

Characteristic	Unmatched				Matched			
	ALL (*n*=272)	Non-surgery (*n*=189)	Surgery (*n*=83)	*P*-value	ALL (*n*=152)	Non-surgery (*n*=76)	Surgery (*n*=76)	*P*-value
Age, years	80.1±4.52	80.6±4.85	78.9±3.40	0.001	79.4±4.07	79.8±4.58	79.1±3.48	0.311
Gender				0.353				0.871
Female	154(56.6%)	111(58.7%)	43(51.8%)		82(53.9%)	40(52.6%)	42(55.3%)	
Male	118(43.4%)	78(41.3%)	40(48.2%)		70(46.1%)	36(47.4%)	34(44.7%)	
Ethnicity				0.432				0.364
White	216(79.4%)	153(81.0%)	63(75.9%)		110(72.4%)	52(68.4%)	58(76.3%)	
Non-white	56(20.6%)	36(19.0%)	20(24.1%)		42(27.6%)	24(31.6%)	18(23.7%)	
Grade				0.015				0.86
Well-moderately	169(62.1%)	108(57.1%)	61(73.5%)		106(69.7%)	52(68.4%)	54(71.1%)	
Poorly-undifferentially	103(37.9%)	81(42.9%)	22(26.5%)		46(30.3%)	24(31.6%)	22(28.9%)	
TNM stage				0.28				0.381
IA	43(15.8%)	24(12.7%)	19(22.9%)		22(14.5%)	7(9.21%)	15(19.7%)	
IB	56(20.6%)	42(22.2%)	14(16.9%)		30(19.7%)	17(22.4%)	13(17.1%)	
II	89(32.7%)	63(33.3%)	26(31.3%)		55(36.2%)	30(39.5%)	25(32.9%)	
IIIA	3(1.10%)	2(1.06%)	1(1.20%)		2(1.32%)	1(1.32%)	1(1.32%)	
IIIB	81(29.8%)	58(30.7%)	23(27.7%)		43(28.3%)	21(27.6%)	22(28.9%)	
Chemotherapy				0.001				1
No	193(71.0%)	122(64.6%)	71(85.5%)		128(84.2%)	64(84.2%)	64(84.2%)	
Yes	79(29.0%)	67(35.4%)	12(14.5%)		24(15.8%)	12(15.8%)	12(15.8%)	
Radiotherapy				0.001				0.442
No	222(81.6%)	144(76.2%)	78(94.0%)		145(95.4%)	74(97.4%)	71(93.4%)	
Yes	50(18.4%)	45(23.8%)	5(6.02%)		7(4.61%)	2(2.63%)	5(6.58%)	

Abbreviations: ICC, intrahepatic cholangiocarcinoma; TNM, tumor node metastasis.

TableS5 Estimated 3-month and 6-month survival rates of surgery and non-surgery groups for M0 stage ICC patients in different age stratification

	Above 60 years old				Above 65 years old				Above 70 years old				Above 75 years old			
	3-Month		6-Month		3-Month		6-Month		3-Month		6-Month		3-Month		6-Month	
	Estimated rate (%)	*P*-value	Estimated rate (%)	*P* -value	Estimated rate (%)	*P* -value	Estimated rate (%)	*P* -value	Estimated rate (%)	*P* -value	Estimated rate (%)	*P* -value	Estimated rate (%)	*P* -value	Estimated rate (%)	*P* -value
OS		<0.001		<0.001		<0.001		<0.001		<0.001		<0.001		<0.001		<0.001
Non-surgery	67.7(62.2-73.8)		53.8(48.0-60.5)		62.1(55.7-69.4)		48.4(41.8-56.1)		59.5(51.9-68.2)		45.2(37.6-54.2)		47.8(37.7-60.7)		35.5(26.1-48.3)	
Surgery	95.2(0.926-0.979)		86.7(82.6-91.0)		93.3(89.8-96.9)		84.5(79.6-89.8)		92.3(88.0-96.8)		82.4(76.4-88.9)		92.1(86.2-98.4)		80.3(71.8-89.7)	
CSS		<0.001		<0.001		<0.001		<0.001		<0.001		<0.001		<0.001		<0.001
Non-surgery	68.1(62.5-74.1)		54.9(49.0-61.5)		62.6(56.1-69.8)		50.3(43.7-57.9)		61.3(53.8-70.0)		47.3(39.7-56.5)		51.3(41.0-64.1)		43.7(33.5-56.9)	
Surgery	95.6(93.0-98.2)		87.9(83.9-92.0)		93.8(90.4-97.3)		85.5(80.7-90.6)		92.9(88.8-97.2)		84.4(78.6-90.1)		92.1(86.2-98.4)		80.3(71.8-89.7)	

TableS6 Estimated 3-month and 6-month survival rates of surgery and non-surgery groups for N0 stage ICC patients in different age stratification

	Above 60 years old				Above 65 years old				Above 70 years old				Above 75 years old			
	3-Month		6-Month		3-Month		6-Month		3-Month		6-Month		3-Month		6-Month	
	Estimated rate (%)	*P*-value	Estimated rate (%)	*P*-value	Estimated rate (%)	*P*-value	Estimated rate (%)	*P*-value	Estimated rate (%)	*P*-value	Estimated rate (%)	*P*-value	Estimated rate (%)	*P*-value	Estimated rate (%)	*P*-value
OS		<0.001		<0.001		<0.001		<0.001		<0.001		<0.001		<0.001		<0.001
Non-surgery	64.6(59.0-70.9)		54.1(48.3-60.7)		61.3(54.8-68.7)		47.5(40.8-55.2)		62.6(54.2-72.4)		50.6(42.0-60.9)		50.4(39.3-64.8)		38.7(28.1-53.2)	
Surgery	96.4(94.1-98.7)		90.0(86.3-93.8)		95.8(93.0-98.7)		87.9(83.3-92.6)		94.5(90.4-98.9)		86.4(80.2-93.0)		93.4(87.4-99.9)		85.2(76.8-94.6)	
CSS		<0.001		<0.001		<0.001		<0.001		<0.001		<0.001		<0.001		<0.001
Non-surgery	65.0(59.4-71.2)		56.0(50.2-62.6)		61.3(54.8-68.7)		49.5(42.8-57.2)		62.6(54.2-72.4)		53.2(44.6-63.5)		50.4(39.3-64.8)		40.1(29.4-54.7)	
Surgery	96.8(94.6-99.0)		91.1(87.7-94.7)		96.3(93.6-99.0)		89.4(85.1-93.9)		94.5(90.4-98.9)		87.3(81.3-93.7)		93.4(87.4-99.9)		85.2(76.8-94.6)	

TableS7 Estimated 3-month and 6-month survival rates of surgery and non-surgery groups for N1 stage ICC patients in different age stratification

	Above 60 years old				Above 65 years old				Above 70 years old				Above 75 years old			
	3-Month		6-Month		3-Month		6-Month		3-Month		6-Month		3-Month		6-Month	
	Estimated rate (%)	*P*-value	Estimated rate (%)	*P* -value	Estimated rate (%)	*P* -value	Estimated rate (%)	*P* -value	Estimated rate (%)	*P* -value	Estimated rate (%)	*P* -value	Estimated rate (%)	*P* -value	Estimated rate (%)	*P* -value
OS		<0.001		<0.001		<0.001		<0.001		<0.001		<0.001		<0.001		<0.001
Non-surgery	63.4(53.0-76.0)		57.5(46.8-70.5)		57.6(45.1-73.6)		51.0(38.5-67.6)		59.7(48.8-73.0)		47.1(36.3-61.1)		55.2(41.7-72.9)		42.6(29.7-61.1)	
Surgery	88.4(81.2-96.3)		73.7(64.0-84.9)		85.4(76.0-96.0)		75.0(63.7-88.3)		86.2(74.5-99.7)		72.4(57.8-90.7)		78.6(59.8-100.0)		64.3(43.5-95.0)	
CSS		<0.001		<0.001		<0.001		<0.001		<0.001		<0.001		<0.001		<0.001
Non-surgery	67.2(56.8-79.5)		60.9(50.1-73.9)		63.0(50.5-78.7)		58.3(45.5-74.6)		64.7(53.8-77.8)		52.8(41.6-67.0)		63.2(49.5-80.7)		51.7(37.8-70.8)	
Surgery	88.4(81.2-96.3)		75.1(65.5-86.1)		85.4(76.0-96.0)		77.0(65.9-89.9)		86.2(74.5-99.7)		75.6(61.3-93.1)		78.6(59.8-100.0)		64.3(43.5-95.0)	

TableS8 Univariate and multivariate cox results table for above 60 years old patients of ICC for overall survival

	No event	Event	Univariable		Multivariable	
	N=152	*N=346*	*HR (95%CI)*	*p-value*	*HR (95%CI)*	*p-value*
TNM:						
IA	41 (27.0%)	36 (10.4%)	Ref.	Ref.	Ref.	Ref.
IB	24 (15.8%)	46 (13.3%)	1.76 [1.14;2.73]	0.011	1.40[0.90;2.17]	0.136
II	48 (31.6%)	134 (38.7%)	2.29 [1.58;3.32]	<0.001	2.24[1.55;3.25]	<0.001
IIIA	3 (1.97%)	8 (2.31%)	2.22 [1.03;4.79]	0.041	1.68[0.78;3.64]	0.186
IIIB	36 (23.7%)	122 (35.3%)	2.58 [1.78;3.75]	<0.001	2.68[1.83;3.91]	<0.001
Radiotherapy:						
No	125 (82.2%)	305 (88.2%)	Ref.	Ref.	Ref.	Ref.
Yes	27 (17.8%)	41 (11.8%)	0.68 [0.49;0.94]	0.02	0.71[0.51;0.99]	0.043
Chemotherapy:						
No	77 (50.7%)	196 (56.6%)	Ref.	Ref.	Ref.	Ref.
Yes	75 (49.3%)	150 (43.4%)	0.70 [0.56;0.87]	0.001	0.51[0.40;0.64]	<0.001
Grade:						
Well-Moderately	103 (67.8%)	203 (58.7%)	Ref.	Ref.	Ref.	Ref.

Poorly-Undifferentially	49 (32.2%)	143 (41.3%)	1.26 [1.02;1.56]	0.034	1.28[1.03;1.59]	0.024
Gender:						
Female	73 (48.0%)	185 (53.5%)	Ref.	Ref.		
Male	79 (52.0%)	161 (46.5%)	0.95 [0.77;1.18]	0.661		
Surgery:						
No	32 (21.1%)	217 (62.7%)	Ref.	Ref.	Ref.	Ref.
Yes	120 (78.9%)	129 (37.3%)	0.28 [0.22;0.35]	<0.001	0.258[0.21;0.32]	<0.001
Race:						
White	127 (83.6%)	269 (77.7%)	Ref.	Ref.		
Non-White	25 (16.4%)	77 (22.3%)	1.24 [0.96;1.60]	0.094		

TableS9 Univariate and multivariate cox results table for above 65 years old patients of ICC for overall survival

	No event	Event	Univariable		Multivariable	
	N=115	*N=273*	*HR (95%CI)*	*p-value*	*HR (95%CI)*	*p-value*
TNM:						
IA	30 (26.1%)	29 (10.6%)	Ref.	Ref.	Ref.	Ref.
IB	19 (16.5%)	43 (15.8%)	1.69 [1.06;2.71]	0.011	1.40[0.87;2.26]	0.163
II	35 (30.4%)	101 (37.0%)	2.18 [1.44;3.30]	<0.001	1.99[1.31;3.04]	0.001
IIIA	2 (1.74%)	7 (2.56%)	2.13 [0.93;4.87]	0.041	1.30[0.56;3.02]	0.537
IIIB	29 (25.2%)	93 (34.1%)	2.31 [1.52;3.52]	<0.001	2.52[1.64;3.88]	<0.001
Radiotherapy:						
No	89 (77.4%)	242 (88.6%)	Ref.	Ref.	Ref.	Ref.
Yes	26 (22.6%)	31 (11.4%)	0.53 [0.36;0.77]	0.001	0.51[0.35;0.75]	<0.001
Chemotherapy:						
No	62 (53.9%)	177 (64.8%)	Ref.	Ref.	Ref.	Ref.
Yes	53 (46.1%)	96 (35.2%)	0.60 [0.47;0.77]	<0.001	0.40[0.31;0.53]	<0.001
Grade:						
Well-Moderately	81 (70.4%)	167 (61.2%)	Ref.	Ref.	Ref.	Ref.
Poorly-Undifferentially	34 (29.6%)	106 (38.8%)	1.42 [1.11;1.81]	0.005	1.54[1.20;1.98]	<0.001
Gender:						

Female	58 (50.4%)	140 (51.3%)	Ref.	Ref.		
Male	57 (49.6%)	133 (48.7%)	1.08 [0.85;1.38]	0.506		
Surgery:						
No	27 (23.5%)	167 (61.2%)	Ref.	Ref.	Ref.	Ref.
Yes	88 (76.5%)	106 (38.8%)	0.29 [0.23;0.38]	<0.001	0.227[0.17;0.30]	<0.001
Race:						
White	94 (81.7%)	219 (80.2%)	Ref.	Ref.		
Non-White	21 (18.3%)	54 (19.8%)	1.11 [0.82;1.50]	0.49		

TableS10 Univariate and multivariate cox results table for above 70 years old patients of ICC for overall survival

	No event	Event	Univariable		Multivariable	
	N=79	*N=205*	*HR (95%CI)*	*p-value*	*HR (95%CI)*	*p-value*
TNM:						
IA	21 (26.6%)	22 (10.7%)	Ref.	Ref.	Ref.	Ref.
IB	12 (15.2%)	38 (18.5%)	1.78 [1.05;3.01]	0.031	1.41[0.83;2.40]	0.2
II	26 (32.9%)	82 (40.0%)	2.25 [1.40;3.61]	0.001	1.79[1.11;2.88]	0.02
IIIA	0 (0.00%)	3 (1.46%)	3.80 [1.13;12.8]	0.031	1.54[0.45;5.22]	0.49
IIIB	20 (25.3%)	60 (29.3%)	2.30 [1.40;3.76]	0.001	2.60[1.58;4.29]	<0.001
Radiotherapy:						
No	68 (86.1%)	187 (91.2%)	Ref.	Ref.		
Yes	11 (13.9%)	18 (8.78%)	0.66 [0.41;1.08]	0.096		
Chemotherapy:						
No	48 (60.8%)	143 (69.8%)	Ref.	Ref.	Ref.	Ref.
Yes	31 (39.2%)	62 (30.2%)	0.66 [0.49;0.89]	0.007	0.46[0.33;0.63]	<0.001
Grade:						
Well-Moderately	52 (65.8%)	126 (61.5%)	Ref.	Ref.		
Poorly-Undifferentially	27 (34.2%)	79 (38.5%)	1.11 [0.84;1.48]	0.455		
Gender:						

Female	38 (48.1%)	99 (48.3%)	Ref.	Ref.		
Male	41 (51.9%)	106 (51.7%)	1.06 [0.81;1.40]	0.655		
Surgery:						
No	17 (21.5%)	125 (61.0%)	Ref.	Ref.	Ref.	Ref.
Yes	62 (78.5%)	80 (39.0%)	0.27 [0.20;0.36]	<0.001	0.23[0.17;0.32]	<0.001
Race:						
White	67 (84.8%)	163 (79.5%)	Ref.	Ref.		
Non-White	12 (15.2%)	42 (20.5%)	1.23 [0.87;1.72]	0.24		

TableS11 Univariate and multivariate cox results table for above 75 years old patients of ICC for overall survival

	No event	Event	Univariable		Multivariable	
	N=37	*N=115*	*HR (95%CI)*	*p-value*	*HR (95%CI)*	*p-value*
TNM:						
IA	11 (29.7%)	11 (9.57%)	Ref.	Ref.	Ref.	Ref.
IB	8 (21.6%)	22 (19.1%)	1.78 [0.86;3.68]	0.118	1.32[0.64;2.74]	0.452
II	10 (27.0%)	45 (39.1%)	2.50 [1.29;4.85]	0.007	1.81[0.93;3.54]	0.081
IIIA	0 (0.00%)	2 (1.74%)	2.35 [0.52;10.6]	0.268	0.77[0.17;3.56]	0.737
IIIB	8 (21.6%)	35 (30.4%)	2.83 [1.42;5.62]	0.003	2.49[1.24;4.98]	0.01
Radiotherapy:						
No	34 (91.9%)	111 (96.5%)	Ref.	Ref.		
Yes	3 (8.11%)	4 (3.48%)	0.54 [0.20;1.48]	0.232		
Chemotherapy:						
No	28 (75.7%)	100 (87.0%)	Ref.	Ref.	Ref.	Ref.
Yes	9 (24.3%)	15 (13.0%)	0.57 [0.33;0.98]	0.044	0.47[0.27;0.82]	0.008
Grade:						
Well-Moderately	29 (78.4%)	77 (67.0%)	Ref.	Ref.		
Poorly-Undifferentially	8 (21.6%)	38 (33.0%)	1.37 [0.93;2.02]	0.116		
Gender:						

Female	21 (56.8%)	61 (53.0%)	Ref.	Ref.		
Male	16 (43.2%)	54 (47.0%)	1.11 [0.77;1.60]	0.579		
Surgery:						
No	6 (16.2%)	70 (60.9%)	Ref.	Ref.	Ref.	Ref.
Yes	31 (83.8%)	45 (39.1%)	0.24 [0.16;0.36]	<0.001	0.22[0.15;0.33]	<0.001
Race:						
White	29 (78.4%)	81 (70.4%)	Ref.	Ref.		
Non-White	8 (21.6%)	34 (29.6%)	1.42 [0.95;2.13]	0.085		

TableS12 Univariate and multivariate cox results table for above 60 years old patients of ICC for cancer-specific survival

	No event	Event	Univariable		Multivariable	
	N=169	*N=329*	*HR (95%CI)*	*p-value*	*HR (95%CI)*	*p-value*
TNM:						
IA	44 (26.0%)	33 (10.0%)	Ref.	Ref.	Ref.	Ref.
IB	26 (15.4%)	44 (13.4%)	1.84 [1.17;2.88]	0.008	1.46[0.93;2.30]	0.102
II	54 (32.0%)	128 (38.9%)	2.37 [1.62;3.48]	<0.001	2.31[1.57;3.39]	<0.001
IIIA	3 (1.78%)	8 (2.43%)	2.40 [1.11;5.21]	0.027	1.80[0.83;3.93]	0.138
IIIB	42 (24.9%)	116 (35.3%)	2.65 [1.80;3.92]	<0.001	2.69[1.81;3.99]	<0.001
Radiotherapy:						
No	141 (83.4%)	289 (87.8%)	Ref.	Ref.	Ref.	Ref.
Yes	28 (16.6%)	40 (12.2%)	0.70 [0.50;0.98]	0.035	0.73[0.52;1.02]	0.065
Chemotherapy:						
No	91 (53.8%)	182 (55.3%)	Ref.	Ref.	Ref.	Ref.
Yes	78 (46.2%)	147 (44.7%)	0.74 [0.59;0.92]	0.006	0.53[0.42;0.68]	<0.001
Grade:						
Well-Moderately	116 (68.6%)	190 (57.8%)	Ref.	Ref.	Ref.	Ref.
Poorly-Undifferentially	53 (31.4%)	139 (42.2%)	1.31 [1.05;1.63]	0.017	1.33[1.07;1.66]	0.011
Gender:						

Female	82 (48.5%)	176 (53.5%)	Ref.	Ref.		
Male	87 (51.5%)	153 (46.5%)	0.95 [0.77;1.18]	0.663		
Surgery:						
No	36 (21.3%)	213 (64.7%)	Ref.	Ref.	Ref.	Ref.
Yes	133 (78.7%)	116 (35.3%)	0.26 [0.20;0.32]	<0.001	0.24[0.19;0.30]	<0.001
Race:						
White	141 (83.4%)	255 (77.5%)	Ref.	Ref.		
Non-White	28 (16.6%)	74 (22.5%)	1.26 [0.97;1.63]	0.085		

TableS13 Univariate and multivariate cox results table for above 65 years old patients of ICC for cancer-specific survival

	No event	Event	Univariable		Multivariable	
	N=130	*N=258*	*HR (95%CI)*	*p-value*	*HR (95%CI)*	*p-value*
TNM:						
IA	33 (25.4%)	26 (10.1%)	Ref.	Ref.	Ref.	Ref.
IB	22 (16.9%)	40 (15.5%)	1.75 [1.07;2.87]	0.026	1.46[0.89;2.41]	0.138
II	41 (31.5%)	95 (36.8%)	2.28 [1.48;3.52]	<0.001	2.08[1.34;3.24]	0.001
IIIA	2 (1.54%)	7 (2.71%)	2.36 [1.02;5.44]	0.044	1.44[0.61;3.36]	0.404
IIIB	32 (24.6%)	90 (34.9%)	2.48 [1.60;3.85]	<0.001	2.67[1.70;4.19]	<0.001
Radiotherapy:						
No	104 (80.0%)	227 (88.0%)	Ref.	Ref.	Ref.	Ref.
Yes	26 (20.0%)	31 (12.0%)	0.57 [0.39;0.82]	0.003	0.55[0.37;0.80]	0.002
Chemotherapy:						
No	73 (56.2%)	166 (64.3%)	Ref.	Ref.	Ref.	Ref.
Yes	57 (43.8%)	92 (35.7%)	0.61 [0.48;0.79]	<0.001	0.41[0.31;0.54]	<0.001
Grade:						
Well-Moderately	93 (71.5%)	155 (60.1%)	Ref.	Ref.	Ref.	Ref.
Poorly-Undifferentially	37 (28.5%)	103 (39.9%)	1.48 [1.15;1.90]	0.002	1.61[1.25;2.08]	<0.001
Gender:						

Female	69 (53.1%)	129 (50.0%)	Ref.	Ref.		
Male	61 (46.9%)	129 (50.0%)	1.14 [0.89;1.45]	0.309		
Surgery:						
No	32 (24.6%)	162 (62.8%)	Ref.	Ref.	Ref.	Ref.
Yes	98 (75.4%)	96 (37.2%)	0.28 [0.21;0.36]	<0.001	0.22[0.16;0.29]	<0.001
Race:						
White	107 (82.3%)	206 (79.8%)	Ref.	Ref.		
Non-White	23 (17.7%)	52 (20.2%)	1.13 [0.83;1.53]	0.43		

TableS14 Univariate and multivariate cox results table for above 70 years old patients of ICC for cancer-specific survival

	No event	Event	Univariable		Multivariable	
	N=97	*N=187*	*HR (95%CI)*	*p-value*	*HR (95%CI)*	*p-value*
TNM:						
IA	26 (26.8%)	17 (9.09%)	Ref.	Ref.	Ref.	Ref.
IB	14 (14.4%)	36 (19.3%)	2.18 [1.22;3.88]	0.008	1.69[0.95;3.03]	0.075
II	32 (33.0%)	76 (40.6%)	2.66 [1.57;4.51]	<0.001	2.08[1.22;3.54]	0.007
IIIA	0 (0.00%)	3 (1.60%)	4.77 [1.39;16.4]	0.013	1.86[0.54;6.46]	0.327
IIIB	25 (25.8%)	55 (29.4%)	2.69 [1.56;4.65]	<0.001	3.01[1.73;5.24]	<0.001
Radiotherapy:						
No	86 (88.7%)	169 (90.4%)	Ref.	Ref.		
Yes	11 (11.3%)	18 (9.63%)	0.74 [0.45;1.20]	0.216		
Chemotherapy:						
No	62 (63.9%)	129 (69.0%)	Ref.	Ref.	Ref.	Ref.
Yes	35 (36.1%)	58 (31.0%)	0.69 [0.51;0.94]	0.019	0.47[0.34;0.65]	<0.001
Grade:						
Well-Moderately	65 (67.0%)	113 (60.4%)	Ref.	Ref.		
Poorly-Undifferentially	32 (33.0%)	74 (39.6%)	1.16 [0.86;1.55]	0.327		
Gender:						

Female	50 (51.5%)	87 (46.5%)	Ref.	Ref.		
Male	47 (48.5%)	100 (53.5%)	1.13 [0.85;1.51]	0.396		
Surgery:						
No	22 (22.7%)	120 (64.2%)	Ref.	Ref.	Ref.	Ref.
Yes	75 (77.3%)	67 (35.8%)	0.24 [0.17;0.33]	<0.001	0.21[0.34;0.65]	<0.001
Race:						
White	82 (84.5%)	148 (79.1%)	Ref.	Ref.		
Non-White	15 (15.5%)	39 (20.9%)	1.25 [0.88;1.78]	0.22		

TableS15 Univariate and multivariate cox results table for above 75 years old patients of ICC for cancer-specific survival

	No event	Event	Univariable		Multivariable	
	N=49	*N=103*	*HR (95%CI)*	*p-value*	*HR (95%CI)*	*p-value*
TNM:						
IA	12 (24.5%)	10 (9.71%)	Ref.	Ref.	Ref.	Ref.
IB	8 (16.3%)	22 (21.4%)	1.96 [0.93;4.15]	0.077	1.48[0.70;3.14]	0.31
II	15 (30.6%)	40 (38.8%)	2.41 [1.20;4.84]	0.013	1.76[0.87;3.55]	0.115
IIIA	0 (0.00%)	2 (1.94%)	2.58 [0.56;11.8]	0.223	0.88[0.19;4.11]	0.868
IIIB	14 (28.6%)	29 (28.2%)	2.52 [1.22;5.21]	0.013	2.20[1.05;4.58]	0.036
Radiotherapy:						
No	46 (93.9%)	99 (96.1%)	Ref.	Ref.		
Yes	3 (6.12%)	4 (3.88%)	0.62 [0.23;1.69]	0.351		
Chemotherapy:						
No	38 (77.6%)	90 (87.4%)	Ref.	Ref.	Ref.	Ref.
Yes	11 (22.4%)	13 (12.6%)	0.55 [0.31;0.99]	0.047	0.46[0.25;0.83]	0.01
Grade:						
Well-Moderately	37 (75.5%)	69 (67.0%)	Ref.	Ref.		
Poorly-Undifferentially	12 (24.5%)	34 (33.0%)	1.35 [0.89;2.04]	0.155		
Gender:						

Female	28 (57.1%)	54 (52.4%)	Ref.	Ref.		
Male	21 (42.9%)	49 (47.6%)	1.13 [0.77;1.66]	0.542		
Surgery:						
No	13 (26.5%)	63 (61.2%)	Ref.	Ref.	Ref.	Ref.
Yes	36 (73.5%)	40 (38.8%)	0.24 [0.16;0.37]	<0.001	0.23[0.25;0.83]	<0.001
Race:						
White	37 (75.5%)	73 (70.9%)	Ref.	Ref.		
Non-White	12 (24.5%)	30 (29.1%)	1.38 [0.90;2.12]	0.139		

TableS16 Estimated 2-year survival rates of surgery and non-surgery groups for ICC patients above 80 years old

		Above 80 years old	
		2-Year	
		Estimated rate (%)	*P*-value
OS			<0.01
	Non-surgery	5.8(0.6-10.9)	
	Surgery	50.4(61.0-39.8)	
CSS			<0.01
	Non-surgery	8.3(1.1-15.5)	
	Surgery	50.4(61.0-39.8)	

TableS17 Estimated 3-month and 6-month survival rates of surgery and non-surgery groups for ICC patients above 80 years old

	M0				N0			
	3-Month		6-Month		3-Month		6-Month	
	Estimated rate (%)	*P*-value	Estimated rate (%)	*P* -value	Estimated rate (%)	*P* -value	Estimated rate (%)	*P* -value
OS		<0.001		<0.001		<0.001		<0.001
Non-surgery	53.9(44.1-63.63)		26.9(18.2-35.6)		58.0(47.3-68.7)		24.1(14.7-33.48)	
Surgery	92.3(87.1-97.5)		84.6(76.8-91.7)		95.5(91.1-99.9)		86.4(79.1-93.7)	
CSS		<0.001		<0.001		<0.001		<0.001
Non-surgery	56.4(46.5-66.3)		38.7(28.7-48.7)		58.0(47.3-68.7)		29.0(19.1-38.9)	
Surgery	92.3(87.1-97.5)		84.6(77.5-91.7)		95.5(91.1-99.9)		86.4(79.1-93.7)	

Figure S1 Flowchart of Patient Selection.

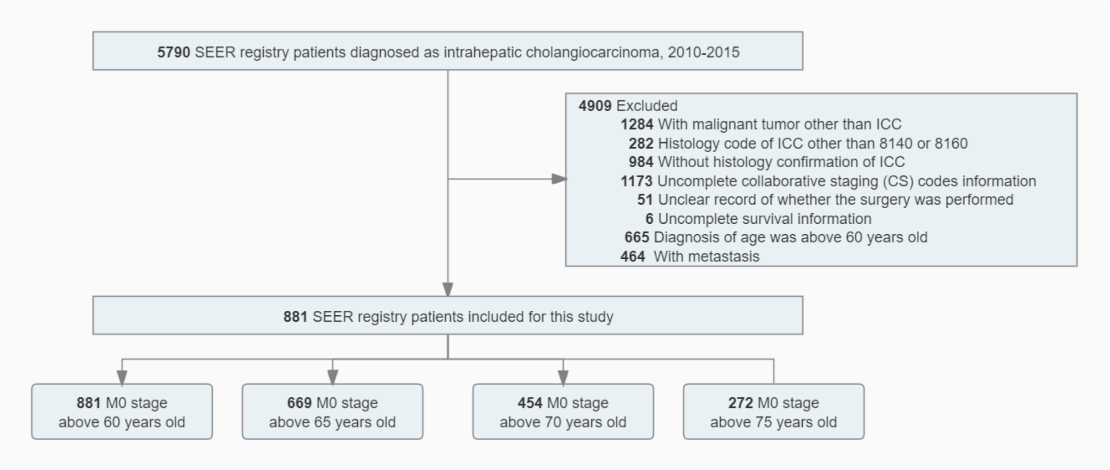

SEER, Surveillance, Epidemiology, and End Results; ICC, intrahepatic cholangiocarcinoma; M0, non-distant metastasis; N0, lymph node-negative; N1, lymph node-positive.

FigureS2 Forest plot and summary statistics of the surgery influence on intrahepatic cholangiocarcinoma incidence for OS in ICC.

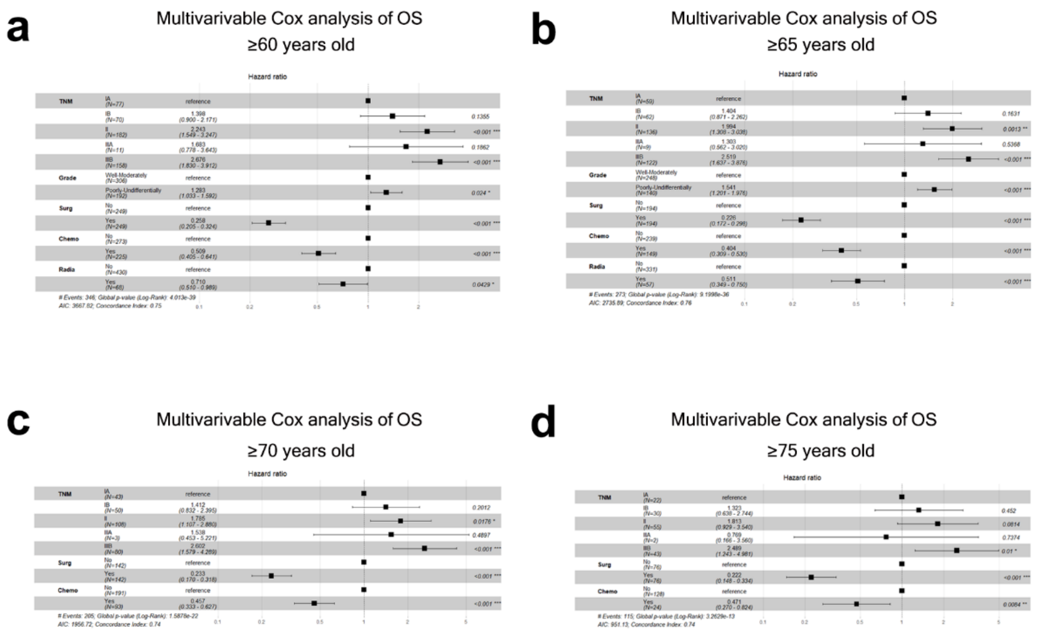

Multivariable Cox regression analysis for OS above 60 years old (a), above 65 years old (b), above 70 years old (c) and above 75 years (d). Abbreviations: ICC, intrahepatic cholangiocarcinoma; OS, overall survival.

FigureS3 Forest plot and summary statistics of the surgery influence on intrahepatic cholangiocarcinoma incidence for CSS in ICC.

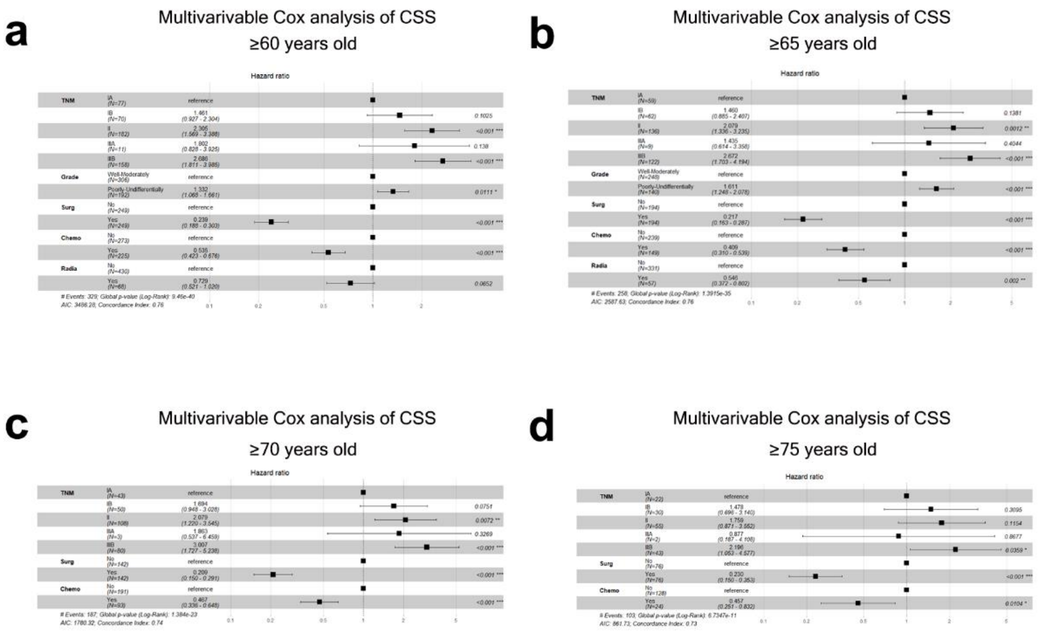

Multivariable Cox regression analysis for OS above 60 years old (a), above 65 years old (b), above 70 years old (c) and above 75 years (d). Abbreviations: ICC, intrahepatic cholangiocarcinoma; CSS cancer-specific survival.
